# Amaryllidaceae Alkaloids as Potential Glycogen Synthase Kinase-3β Inhibitors

**DOI:** 10.3390/molecules23040719

**Published:** 2018-03-21

**Authors:** Daniela Hulcová, Kateřina Breiterová, Tomáš Siatka, Kamila Klímová, Lara Davani, Marcela Šafratová, Anna Hošťálková, Angela De Simone, Vincenza Andrisano, Lucie Cahlíková

**Affiliations:** 1 ADINACO Research Group, Department of Pharmacognosy, Faculty of Pharmacy, Charles University, 500 05 Hradec Králové, Czech Republic; hulcovd@faf.cuni.cz (D.H.); siatka@faf.cuni.cz (T.S.); safratom@faf.cuni.cz (M.Š.); 2 Department of Pharmaceutical Botany, Faculty of Pharmacy, Charles University, 500 05 Hradec Králové, Czech Republic; breiterk@faf.cuni.cz (K.B.); kklimova000@gmail.com (K.K.); hosta4aa@faf.cuni.cz (A.H.); 3 Department for Life Quality Studies, University of Bologna, 47921 Rimini, Italy; lorisdavani@libero.it (L.D.); angela.desimone2@unibo.it (A.D.S.); vincenza.andrisano@unibo.it (V.A.)

**Keywords:** Amaryllidaceae alkaloids, Alzheimer’s disease, glycogen synthase kinase-3β, masonine, caranine, 9-*O*-demethylhomolycorine

## Abstract

Glycogen synthase kinase-3β (GSK-3β) is a multifunctional serine/threonine protein kinase that was originally identified as an enzyme involved in the control of glycogen metabolism. It plays a key role in diverse physiological processes including metabolism, the cell cycle, and gene expression by regulating a wide variety of well-known substances like glycogen synthase, tau-protein, and β-catenin. Recent studies have identified GSK-3β as a potential therapeutic target in Alzheimer´s disease, bipolar disorder, stroke, more than 15 types of cancer, and diabetes. GSK-3β is one of the most attractive targets for medicinal chemists in the discovery, design, and synthesis of new selective potent inhibitors. In the current study, twenty-eight Amaryllidaceae alkaloids of various structural types were studied for their potency to inhibit GSK-3β. Promising results have been demonstrated by alkaloids of the homolycorine-{9-*O*-demethylhomolycorine (IC_50_ = 30.00 ± 0.71 µM), masonine (IC_50_ = 27.81 ± 0.01 μM)}, and lycorine-types {caranine (IC_50_ = 30.75 ± 0.04 μM)}.

## 1. Introduction

Glycogen synthase kinase-3β (GSK-3β) is a ubiquitous pleiotropic serine/threonine kinase that plays crucial roles in cellular functions, including cell-cycle regulation, differentiation, and proliferation, and gene expression by regulating a wide variety of known targets such as glycogen synthase, τ-protein, and β-catenin [1]. GSK-3 is involved in cellular signaling, including Wnt and Hedgehog pathways, and in neuronal development, insulin pathways, transcription, cell division, cell survival, and cell death [1,2,3]. Due to its multifarious roles, aberrant activity of GSK-3 underlines a variety of disorders including Alzheimer´s disease (AD) [4], cancer [5], diabetes [6], cardiovascular disorders [7], and psychiatric disorders [8].

One of the neuropathological characteristics of AD is the presence of neurofibrillary tangles (NFTs) consisting of paired helical filaments, with the main component being hyperphosphorylated τ-protein. Phosphorylation of τ-proteins is primarily dependent on GSK-3β and cyclin-dependent kinase 5 (CDK5) [9]. Genetic and epidemiological studies indicate that GSK-3β is deregulated in AD through alterations in upstream Wnt and insulin signaling pathway intermediates. This may be the reason behind tau hyperphosphorylation and, later on, the formation of NFTs. GSK-3β may also induce the formation of amyloid β-protein (Aβ), a further neuropathological marker for AD. Aβ is aggregated and deposited in the AD brain and causes dysfunction of neurons, inflammation, and oxidative stress [10]. Aβ production is facilitated by overexpression of β-site amyloid precursor protein (APP)-cleaving enzyme 1 (BACE1) and of presenilin 1 (PS1) [11]. Increased GSK-3β activity in the brains of patients with AD, and its pathological activation facilitates Aβ production [12]. Therapeutic concentrations of lithium, a GSK-3 inhibitor, block the production of Aβ peptides and the accumulation of Aβ peptides in the brains of mice that overproduce APP [13,14]. Clinical studies have evaluated the safety and efficacy of the irreversible GSK-3β inhibitor tideglusib in the treatment of patients with AD [15,16]. Tideglusib is a thiadiazolidinone that reduces tau phosphorylation in murine primary neurons. In a pilot, double-blind, placebo-controlled, randomized, escalating dose trial, 30 patients with mild to moderate Alzheimer´s disease were enrolled and received either tideglusib or placebo (orally) at escalating doses for a total of 20 weeks. The objective of this pilot study was to evaluate safety and tolerability of tideglusib with strict criteria for drug escalation or withdrawal. Tideglusib was well tolerated by 65% of the patients [16].

GSK-3β has been implicated in playing a role in cancers which are resistant to chemo-, radio-, and targeted therapy [17]. It has been shown to be a potential mediator in contributing to neoplastic transformation, in part because it belongs to both the canonical Wnt/β-catenin and the PI3K/Akt signaling systems, the two major pathways often dysregulated in cancer [18]. GSK-3 inhibitors may eventually be used in the treatment of certain cancers. GSK-3 is believed to exert pro-proliferative effects in solid cancers including: colorectal cancer, glioblastoma, pancreatic cancer, ovarian cancer, and blood cancers [19]. 

A number of publications have emerged describing diverse molecules that inhibit GSK-3β, such as manzamine alkaloids [20], pyrazolopyrimidines [21], pyridyloxadiazoles [22], thiadiazolidindiones [23], maleimides [24], and paullones (a group of benzazepinones) [25]. Current advances in the search for GSK-3 inhibitors have been recently reviewed [1,13,26].

Amaryllidaceae alkaloids, consisting of a nitrogen-containing polycyclic structure, are produced exclusively by plants of the Amaryllidaceae family. These compounds have attracted considerable attention, most prominently because of their inhibition of acetylcholinesterase (AChE) and activity against drug-resistant cancers with dismal prognoses [27,28,29,30]. The best known Amaryllidaceae alkaloid, galanthamine, is used in the treatment of Alzheimer’s disease, as a long acting, selective, reversible, and competitive AChE inhibitor [28]. Further Amaryllidaceae alkaloids, such as pancratistatine, narciclasine, lycorine, haemanthamine, distichamine, and their derivatives, are known for their potent cell line specific anticancer properties, and some of them are involved at various stages of development, with a clinical candidate earmarked for commercialization within the next decade [31,32].

In our search for active natural products against neurological and cancer disorders, we have discovered the potency of Amaryllidaceae alkaloids to inhibit GSK-3β.

## 2. Results and Discussion

### 2.1. Amaryllidaceae Alkaloids

**Figure 1:**
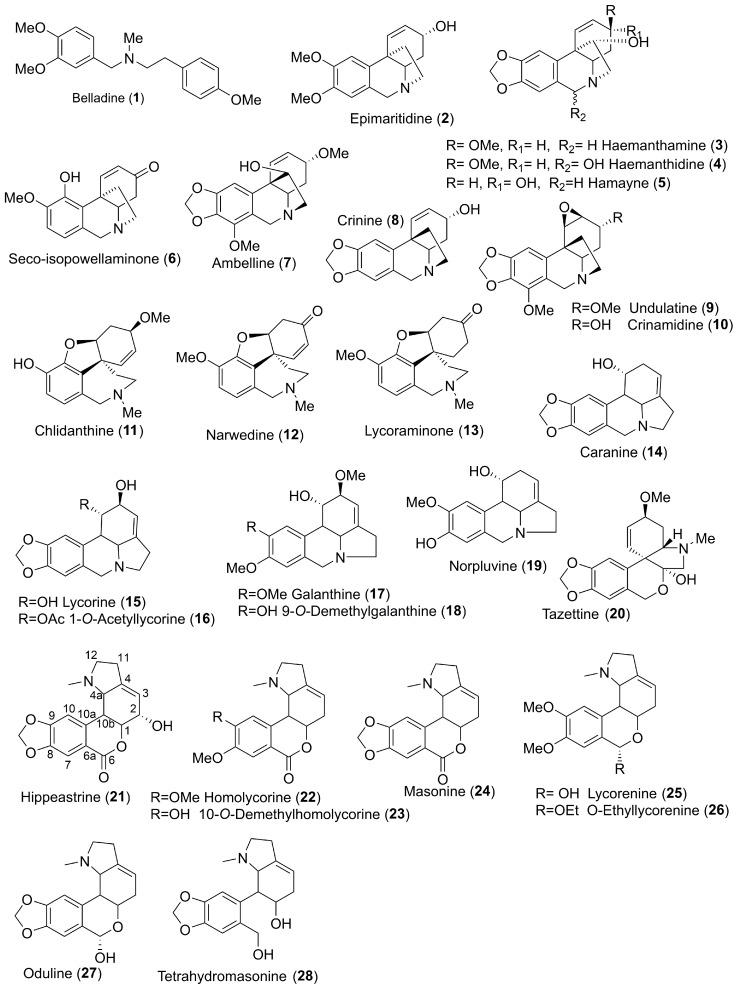
Structures of the studied Amaryllidaceae alkaloids.

In the current study, 28 Amaryllidaceae alkaloids (Figure 1) of seven structural types: belladine (**1**), haemanthamine (**2**–**6**), crinine (**7**–**10**), galanthamine (**11**–**13**), lycorine (**14**–**19**), tazettine (**20**), and homolycorine (**21**–**28**), were studied for their ability to inhibit GSK-3β. All compounds have been previously isolated in our laboratory from different Amaryllidaceae plants.

### 2.2. Potency of Amaryllidaceae Alkaloids to Inhibit GSK-3β

The inhibitory activity of the compounds was first screened at a concentration of 50 µM (Table 1); a synthetic arylindolemaleimide derivative, SB-415286, was used as a positive standard. This compound is a highly selective GSK-3 inhibitor developed by GlaxoSmithKline that inhibits GSK-3 as well as other organic inhibitors of synthetic origin (e.g., thiadiazolidinones, oxadiazole analogues), within the low nanomolar concentration range [23,24,33].

The best results in preliminary screening were demonstrated by alkaloids of the homolycorin-type (**21**–**28**). Most of the substances tested in this group showed an activity at 50 μM of more than 50%. After preliminary screening, the three most active compounds: caranine (**14**), 9-*O*-demethylhomolycorine (**23**), and masonine (**24**), were selected for IC_50_ determination. 

**Figure 2:**
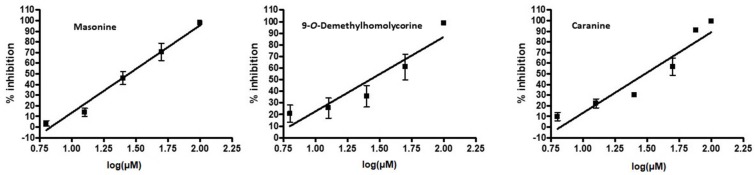
Linear graph of IC_50_ assay of GSK-3β treated with selected Amaryllidaceae alkaloids. Concentrations of alkaloids were 6.25; 12.5; 25; 50 and 100 μM. Activity is presented as % inhibition.

The measurements were performed in triplicate and the values given are the average obtained after at least two measurements. The IC_50_ values of the selected alkaloids are in the micromolar range (about 30 µM) and were obtained for three of the selected compounds (Table 2). The highest GSK-3β inhibition potency has been demonstrated by two homolycorine-type Amaryllidaceae alkaloids, masonine (**24**, IC_50_ = 27.81 ± 0.01 µM; Figure 2) and 9-*O*-demethylhomolycorine (**23**, IC_50_ = 30.00 ± 0.71 µM; Figure 2), and one lycorine-type alkaloid caranine (**14**, IC_50_ = 30.75 ± 0.04 µM; Figure 2). The low number of available homolycorine-type alkaloids precluded a detailed structure-activity relationship (SAR) study, but their general features can still be described. It seems that the presence of hydroxyl substitution at position 2, as in hippeastrine (**21**; see Figure 1), is connected with a distinct reduction of GSK-3β inhibitory activity (10.65% of GSK-3β inhibition at 50 µM) compared with masonine (66.0% of GSK-3β inhibition at 50 µM), 9-*O*-demethylhomolycorine (63.6% of GSK-3β inhibition at 50 µM), oduline (57.7% of GSK-3β inhibition at 50 µM), and *O*-ethyllycorenine (57.7% of GSK-3β inhibition at 50 µM), where no substituent (e.g., hydroxy or methoxy group, etc.) in position C-2 is present. The opening of the tetrahydropyrane ring in tetrahydromasonine (**28,** see Figure 1) also reduces the GSK-3β inhibitory potency of homolycorine-type alkaloids (Table 1). For a detailed SAR study of homolycorine-type of Amaryllidaceae alkaloids, it is necessary to study a wider range of natural or semi-synthetic analogues of active alkaloids.

The most interesting GSK-3β inhibition potency of natural products have been demonstrated by the alkaloid manzamine A (IC_50_ = 10.2 µM), isolated from a common Indonesian sponge *Acanthostrongylophora* and its semisynthetic analogue 1 [20], by indole alkaloid hymenialdisine (HD, IC_50_ = 10 nM) [34], isolated from marine sponges from the Agelasidae, Axinellidae, and Halichondriidae families [35,36], as well as meridianin E (IC_50_ = 2.5 µM) [37] isolated from ascidian *Aplidium meridianum*. The mechanism of action has been studied in case of HD. The kinetic experiments were performed by varying both ATP levels and HD concentrations. The results of double-reciprocal plotting indicated that HD is a competitive inhibitor for ATP [34]. Compounds isolated from endophytic fungus *Cosmospora vilior* have also been studied for their potency to inhibit GSK-3β [38]. Cosmochlorin A and cosmochlorine B showed GSK-3β inhibition activity at IC_50_ values of 62.5 and 60.6 µM, respectively [38].

## 3. Experimental

### 3.1. Amaryllidaceae Alkaloids

All Amaryllidaceae alkaloids tested have been previously isolated at the Department of Pharmaceutical Botany, Faculty of Pharmacy in Hradec Králové from various Amaryllidaceae plant species (*Zephyranthes robusta* [39,40], *Chlidanthus fragrans* [27,41], *Nerine bowdenii* [42], *Narcissus poeticus* cv. Pink Parasol [43], and *N. poeticus* cv. Brackenhurst [44]). The purity of all compounds (≥ 98%) was determined by ^1^H and ^13^C NMR spectroscopy.

### 3.2. GSK-3β Assay

Kinase-Glo Kit was obtained from Promega (Promega Biotech Iberica, S.L., Madrid, Spain), and human recombinant GSK-3β and GSM substrate mimicking Glycogen Muscle Synthase from Merck Millipore (Darmstadt, Germany). Adenosine 5-triphosphate (ATP) disodium salt hydrate, ammonium acetate, ammonium hydroxide, 4-(2-hydroxyethyl)piperazine-1-ethanesulfonic acid (HEPES), ethylene glycol-bis(-aminoethylether)-*N*,*N*,*N*,*N*-tetraacetic acid tetrasodium salt (EGTA), ethylenediaminetetraacetic acid (EDTA), dimethyl sulfoxide (DMSO), magnesium acetate tetrahydrate, formic acid, and 3-[(3-chloro-4-hydroxyphenyl)amino]-4-(2-nitrophenyl)-1H-pyrrol-2,5-dione were purchased from Sigma-Aldrich (St. Louis, MO, USA). The GSK-3β selective inhibitor SB-415286 ([3-(3-chloro-4-hydroxyphenylamino)-4-(2-nitrophenyl)-1H-pyrrole-2,5-dione]) was purchased from Selleck Chemicals (Houston, TX, USA). Ultrapure water was obtained using a Purite LTD water purification system (Thame, UK). The experiments were carried out using a Victor X3 multimode plate reader (Perkin Elmer, MA, USA).

GSK-3β activity and inhibition were studied according to the luminescent method of Baki et al. using a Kinase-Glo reagent kit [45]. The reaction was performed in 96-well white plates. Each well contained 10 µL of test compound (dissolved in DMSO) at 1 mM concentration and diluted in advance in an assay buffer (pH 7.5) containing 50 mM HEPES, 1 mM EDTA, 1 mM EGTA, and 15 mM magnesium acetate, to the desired concentration, 10 µL of ATP (1 µM final concentration), 10 µL of 100 µM GSM and 10 µL of GSK-3β (20 ng). Ten microliters of either buffer or SB-415286 solution (5 µM final concentration) was added instead of test compound solution in order to obtain the positive (maximum activity) and negative control (total inhibition), respectively. The final DMSO concentration in the reaction mixture did not exceed 5%. The mix was left to react at 37 °C for 30 min. Then the enzymatic reactions were stopped with 40 µL of Kinase-Glo reagent. Glow-type luminescence was recorded after 10 min. The activity is proportional to the difference of the total and consumed ATP. The inhibition activities were calculated on the basis of maximal activity, measured in the absence of inhibitor, and the maximal inhibition was measured in the presence of the reference compound. The IC_50_ values were calculated using the GraphPad Prism 4.0 program (GraphPad Software Inc., CA, USA).

## 4. Conclusions

In conclusion, GSK-3β is an enzyme with a very large number of different actions in intracellular signaling systems. Many of the pathways that use GSK-3β as a regulator have links to human diseases and, thus, have great potential as a target for therapeutic prevention. Currently, GSK-3β inhibitors have great promise as drugs for the pharmacotherapy of severe pathologies such as cancer, AD, mood disorders, diabetes, stroke, and many others. Since the introduction of galanthamine into the treatment of AD, Amaryllidaceae alkaloids have been an important source for the discovery of potential therapeutic agents. 

In the present study, the potency of Amaryllidaceae alkaloids to inhibit GSK-3β has been studied. The results obtained suggest Amaryllidaceae alkaloids constitute an interesting scaffold. Since Amaryllidaceae alkaloids can easily be isolated from natural sources in amounts which allow for the preparation of their derivatives, thus the active GSK-3β inhibitors will be used in the design of more potent semisynthetic compounds. The type of GSK-3β inhibition of active alkaloids, and their semisynthetic derivatives, will be studied in future experiments.

## Figures and Tables

**Table 1:** Screening of Amaryllidaceae alkaloids for their potency to inhibit GSK-3β (conc. 50 µM).

Structural Type	Alkaloid	% of Inhibition
Belladine	Beladine (**1**)	34.4 ± 2.7
Haemanthamine	Epimaritidine (**2**)	45.2 ± 1.1
	Haemanthamine (**3**)	52.4 ± 0.1
	Haemanthidine (**4**)	33.0 ± 2.2
	Hamayne (**5**)	33.9 ± 0.1
	Seco-isopowellaminone (**6**)	38.5 ± 0.8
Crinine	Ambelline (**7**)	38.0 ± 0.8
	Crinine (**8**)	39.6 ± 5.4
	Undulatine (**9**)	43.3 ± 4.0
	Crinamidine (**10**)	32.1 ± 7.9
Galanthamine	Chlidanthine (**11**)	37.9 ± 9.5
	Narwedine (**12**)	37.7 ± 0.3
	Lycoraminone (**13**)	38.9 ± 1.0
Lycorine	Caranine (**14**)	61.8 ± 9.2
	Lycorine (**15**)	32.9 ± 0.2
	1-*O*-Acetyllycorine (**16**)	49.9 ± 1.9
	Galanthine (**17**)	26.4 ± 7.7
	9-*O*-Demethylgalanthine (**18**)	50.9 ± 8.9
	Norpluviine (**19**)	45.0 ± 4.3
Tazettine	Tazettine (**20**)	49.2 ± 0.3
Homolycorine	Hippeastrine (**21**)	10.7 ± 2.5
	Homolycorine (**22**)	54.4 ± 0.6
	9-*O*-Demethylhomolycorine (**23**)	63.6 ± 1.3
	Masonine (**24**)	66.0 ± 4.0
	Lycorenine (**25**)	47.6 ± 3.5
	*O*-Ethyllycorenine (**26**)	57.7 ± 3.5
	Oduline (**27**)	57.7 ± 4.4
	Tetrahydromasonine (**28**)	22.4 ± 0.2

**Table 2:** The potency to inhibit GSK-3β (IC_50_) of selected Amaryllidaceae alkaloids.

Alkaloid	IC_50_ (µM) *
Caranine (**14**)	30.75 ± 0.04
9-*O*-Demethylhomolycorine (**23**)	30.00 ± 0.71
Masonine (**24**)	27.81 ± 0.05
SB-415286 **	70.00 nM

* Data are the means ± Standard Deviation (SD) of three independent replications, ** SB-415286, a compound used as a standard.

## References

[1] Saraswati A.P., Ali Hussaini S.M., Krishna N.H., Babu B.N., Kamal A. (2018). Glykogen synthase kinase-3 and its inhibitors: Potential target for various therapeutics conditions. Eur. J. Med. Chem..

[2] Cohen P., Frame S. (2001). The renaissance of GSK3. Nat. Rev. Mol. Cell Biol..

[3] Phukan S., Babu V.S., Kannoji A., Hariharan R., Balaji V.N. (2010). GSK3β: Role in therapeutic landscape and development of modulators. Br. J. Pharmacol..

[4] Maqbool M., Mobashir M., Hoda N. (2016). Pivotal role of glycogen synthase kinase-3: A therapeutic target for Alzheimer’s disease. Eur. J. Med. Chem..

[5] Luo J. (2009). Glycogen synthase kinase 3β (GSK3β) in tumorigenesis and cancer chemotherapy. Cancer Lett..

[6] Henriksen E.J., Dokken B.B. (2006). Role of glycogen synthase kinase-3 in insulin resistance and type 2 diabetes. Curr. Drug Targets.

[7] Lal H., Ahmad F., Woodgett J., Force T. (2015). The GSK-3 family as therapeutic target for myocardial diseases. Circ. Res..

[8] Jope R.S., Roh M.S. (2006). Glykogen synthase kinase-3 (GSK3) in psychiatric diseases and therapeutic interventions. Curr. Drug Targets.

[9] Plattner F., Angelo M., Giese K.P. (2006). The roles of cyclin-dependent kinase 5 and glycogen synthase kinase 3 in tau hyperphosphorylation. J. Biol. Chem..

[10] Palop J.J., Mucke L. (2010). Amyloid-β induced neuronal dysfunction in Alzheimer’s disease: From synapses toward neural networks. Nat. Neurosci..

[11] Petanceska S.S., Seeger M., Checler F. (2000). Mutant presenilin 1 increases the levels of Alzheimer amyloid β-peptide Aβ42 in late compartments of the constitutive secretory pathway. J. Neurochem..

[12] Rockenstein E., Torrance M., Adame A., Mante M., Baron P., Rose J.B., Crews L., Masliah E. (2007). Neuroprotective effects of regulators of the glycogen synthase kinase-3β signaling pathway in a transgenic model of Alzheimer’s disease are associated with reduced amyloid precursor protein phosphorylation. J. Neurosci..

[13] Martinez A. (2008). Preclinical efficacy on GSK-3 inhibitors: Towards a future generation of powerful drugs. Med. Res. Rev..

[14] Phiel C.J., Wilson C.A., Lee V.M., Klein P.S. (2003). GSK-3α regulates production of Alzheimer’s disease amyloid-β peptides. Nature.

[15] Dominguez J.M., Fuertes A., Orozco L., del Monte-Millan M., Deldago E., Medina M. (2012). Evidence for irreversible inhibition of glycogen synthase kinase-3β by tideglusib. J. Biol. Chem..

[16] Del Ser T., Steinwachs K.C., Gertz H.J., Andress M.V., Gomez-Carrillo B., Medina M., Vericat J.A., Redondo P., Fleet D., Leon T. (2013). Treatment of Alzheimer’s disease with the GSK-3 inhibitor tideglusib: A pilot study. J. Alzheimer Dis..

[17] Shimura T. (2011). Acquired radioresistance of cancer and the AKT/GSK3β/cyclin D1 overexpression cycle. J. Radiat. Res..

[18] Jope R.S., Yuskaitis C.J., Beurel E. (2007). Glycogen synthase kinase-3 (GSK3): Inflammation, diseases, and therapeutics. Neurochem. Res..

[19] McCubrey J.A., Steelman L.S., Bertrand F.E., Davis N.M., Sokolosky M., Abrams S.L., Montalto G., D’Assoro A.B., Libra M., Nicoletti F. (2014). GSK-3 as potential target for therapeutic intervention in cancer. Oncotarget.

[20] Hamann M., Alonso D., Martín-Aparicio E., Fuertes A., Pérez-Puerto M.J., Castro A., Morales S., Navarro M.L., Del Monte-Millán M., Medina M. (2007). Glycogen synthase kinase-3 (GSK-3) inhibitory activity and structure-activity relationship (SAR) studies of the manzamine alkaloids. Potential for Alzheimer’s disease. J. Nat. Prod..

[21] Witherington J., Bordas V., Garland S.L., Hickey D.M.B., Ife R.J., Liddle J., Saunders M., Smith D.G., Ward R.W. (2003). 5-Aryl-pyrazolo[3,4-b]pyridines: Potent inhibitors of glycogen synthase kinase-3 (GSK-3). Bioorg. Med. Chem..

[22] Naerum L., Norskov-Lauritsen L., Olesen P.H. (2002). Scaffold hopping and optimization towards libraries of glycogen synthase kinase-3 inhibitors. Bioorg. Med. Chem. Lett..

[23] Martinez A., Alonso M., Castro A., Perez C., Moreno F.J. (2002). First non-ATP competitive glycogen synthase kinase 3 β (GSK-3β) inhibitors:  Thiadiazolidinones (TDZD) as potential drugs for the treatment of Alzheimer’s disease. J. Med. Chem..

[24] Coghlan M.P., Culbert A.A., Cross D.A.E., Corcoran S.L., Yates J.D., Pearce N.J., Rausch O.L., Murphy G.J., Carter P.S., Cox L.R. (2000). Selective small molecule inhibitors of glycogen synthase kinase-3 modulate glycogen metabolism and gene transcription. Chem. Biol..

[25] Leost M., Schultz C., Link A., Wu Y.Z., Biernat J., Man-Delkow E.M., Bibb J.A., Snyder G.L., Greengard P., Zaharevitz D.W. (2000). Paullones are potent inhibitors of glycogen synthase kinase-3β and cyclin-dependent kinase 5/p25. Eur. J. Biochem..

[26] Pandey M.K., DeGrado T.R. (2016). Glycogen synthase kinase-3 (GSK-3)-targeted therapy and imaging. Theranostics.

[27] Doskočil I., Hošťálková A., Šafratová M., Benešová N., Havlík J., Havelek R., Kuneš J., Královec K., Chlebek J., Cahlíková L. (2015). Cytotoxic activities of Amaryllidaceae alkaloids against gastrointestinal cancer cells. Phytochem. Lett..

[28] Ago Y., Koda K., Takuma K., Matsuda T. (2011). Pharmacological aspects of the acetylcholinesterase inhibitor galantamine. J. Pharm. Sci..

[29] Cahlíková L., Pérez D.I., Štěpánková Š., Chlebek J., Šafratová M., Hošťálková A., Opletal L. (2015). In vitro inhibitory effects of 8-*O*-demethylmaritidine and undulatine on acetylcholinesterase and their predicted penetration across the blood-brain barrier. J. Nat. Prod..

[30] Cedrón J.C., Ravelo A.G., León L.G., Padrón J.M., Estévez-Braun A. (2015). Antiproliferative and structure activity relationships of Amaryllidaceae alkaloids. Molecules.

[31] Van Goietsenoven G., Hutton J., Becker J.P., Lallemand B., Robert F., Lefranc F., Pirker C., Vandenbussche G., Van Antwerpen P., Evidente A. (2010). Targeting of eEF1A with Amaryllidaceae isocarbostyrils as a strategy to combat melanomas. FASEB J..

[32] Ma D., Pignanelli C., Tarade D., Gilbert T., Noel M., Mansour F., Adams S., Dowhayko K., Vshyvenko S., Hudlicky T. (2017). Cancer cell mitochondria targeting by pancratistatin analogs is dependent on functional complex II and III. Sci. Rep..

[33] Saitoh M., Kunitomo J., Kimura E., Hayase Y., Kobayashi H., Uchiyama N., Kawamoto T., Tanaka T., Mol C., Dougan D.R. (2009). Design, synthesis and structure-activity relationships of 1,3,4-oxadiazole derivatives as novel inhibitors of glycogen synthase kinase-3β. Bioorg. Med. Chem..

[34] Meijer L., Thunnissen A.-M.W.H., White A.W., Garnier M., Nikolic M., Tsai L.-H., Walter J., Cleverley K.E., Salinas P.C., Wu Y.-Z. (2000). Inhibition of cyclin-dependent kinases, GSK-3β and CK1 by hymenialdisine, a marine sponge constituent. Chem. Biol..

[35] Kitagawa I., Kobayashi M., Kitanaka K., Kido M., Kyogoku Y. (1983). Marine natural products XII. On the chemical constituents of the Okinawan marine sponge *Hymeniacidon aldis*. Chem. Pharm. Bull..

[36] Cimino G., de Rosa S., de Stefano S., Mazzarella L., Puliti R., Sodano G. (1982). Isolation and X-ray crystal structure of a novel bromo-compound from two marine sponges. Tetrahedron Lett..

[37] Gompel M., Leost M., Bal De Kier J.E., Puricelli L., Hernandez F.L., Palermo J., Meijer L. (2004). Meridianins, a new family of protein kinase inhibitors isolated from the Ascidian *Aplidium meridianum*. Bioorg. Med. Chem. Lett..

[38] Shiono Y., Miyazaki N., Murayyma T., Harizon T.K., Katja D.G., Supratman U., Nakata J., Kakihara Y., Saeki M., Yoshida J. (2016). GSK-3β inhibitory activities of novel dichlororesorcinol derivatives from *Cosmopora vilior* isolated from mangrove plant. Phytochem. Lett..

[39] Kulhánková A., Cahlíková L., Novák Z., Macáková K., Kuneš J., Opletal L. (2013). Alkaloids from *Zephyranthes robusta* Baker and their acetylcholinesterase and butyrylcholinesterase-inhibitory activity. Chem. Biodivers..

[40] Šafratová M., Novák Z., Kulhánková A., Kuneš J., Hrabinová M., Jun D., Macáková K., Opletal L., Cahlíková L. (2014). Revised NMR data for 9-*O*-demethylgalanthine: An alkaloid from *Zephyranthes robusta* (Amaryllidaceae) and its biological activity. Nat. Prod. Commun..

[41] Cahlíková L., Hrabinová M., Kulhánková A., Benešová N., Chlebek J., Jun D., Novák Z., Kuča K., Macáková K., Opletal L. (2013). Alkaloids from *Chlidanthus fragrans* and their acetylcholinesterase, butyrylcholinesterase and prolyl oligopeptidase activities. Nat. Prod. Commun..

[42] Vaněčková N., Hošťálková A., Šafratová M., Kuneš J., Hulcová D., Hrabinová M., Doskočil I., Štěpánková Š., Opletal L., Nováková L. (2016). Isolation of Amaryllidaceae alkaloids from *Nerine bowdenii* W. Watson and their biological activities. RSC Adv..

[43] Šafratová M., Hošťálková A., Hulcová D., Breiterová K., Hrabcová V., Machado M., Fontinha D., Prudêncio M., Kuneš J., Chlebek J. (2017). Alkaloids from *Narcissus poeticus* cv. Pink Parasol of various structural types and their biological activity. Arch. Pharm. Res..

[44] Havlasová J., Šafratová M., Siatka T., Štěpánková Š., Ločárek M., Opletal L., Hrabinová M., Jun D., Benešová N., Novák Z. (2014). Chemical composition of bioactive alkaloid extracts from some *Narcissus* species and varieties and their biological activity. Nat. Prod. Commun..

[45] Baki A., Bielik A., Molnár L., Szendrei G., Keserü G.M. (2007). A high throughput luminescent assay for glycogen synthase kinase-3β inhibitors. ASSAY Drug. Dev. Technol..

